# Lipopolysaccharide induces acute bursal atrophy in broiler chicks by activating TLR4-MAPK-NF-κB/AP-1 signaling

**DOI:** 10.18632/oncotarget.19964

**Published:** 2017-08-05

**Authors:** Abdur Rahman Ansari, Ning-Ya Li, Zhi-Jian Sun, Hai-Bo Huang, Xing Zhao, Lei Cui, Ya-Fang Hu, Ju-Ming Zhong, Niel A. Karrow, Hua-Zhen Liu

**Affiliations:** ^1^ Department of Basic Veterinary Medicine, College of Animal Science and Veterinary Medicine, Huazhong Agricultural University, Wuhan, Hubei, China; ^2^ Department of Basic Sciences, Section of Anatomy and Histology, College of Veterinary and Animal Sciences (CVAS) Jhang, University of Veterinary and Animal Sciences (UVAS), Lahore, Pakistan; ^3^ Department of Anatomy, Physiology and Pharmacology, College of Veterinary Medicine, Auburn University, Auburn, USA; ^4^ Department of Animal Biosciences, University of Guelph, Guelph, Ontario, Canada

**Keywords:** lipopolysaccharide, chicken, bursa of Fabricius, acute atrophy, transcriptional analysis, Immunology

## Abstract

We investigated the mechanisms that induce atrophy of the chicken bursa of Fabricius (BF) upon lipopolysaccharide (LPS) treatment in young chicks. LPS treatment resulted in ∼36% decrease in bursal weight within 36 h (*P* < 0.01). Histological analysis showed infiltration of eosinophilic heterophils and nucleated oval shaped RBCs in or near blood vessels of the BF from LPS-treated chicks. Scanning electron micrographs showed severe erosion and breaks in the mucosal membrane at 12 h and complete exuviation of bursal mucosal epithelial cells at 36 h. We observed decreased cell proliferation (low PCNA positivity) and increased apoptosis (high TUNEL and ssDNA positivity) in the BF 12-72 h after LPS treatment. RNA-seq analysis of the BF transcriptome showed 736 differentially expressed genes with most expression changes (637/736) 12 h after LPS treatment. KEGG pathway analysis identified TLR4-MAPK-NF-κB/AP-1 as the key signaling pathway affected in response to LPS stimulation. These findings indicate LPS activates the TLR4-MAPK-NF-κB/AP-1 signaling pathway that mediates acute atrophy of the chicken bursa of Fabricius by inducing inflammation and apoptosis.

## INTRODUCTION

The bursa of Fabricius (BF) is an avian specific immune organ. It has a specialized microenvironment that supports the differentiation of B cells and production of antibodies [1, 2]. Acute atrophy of BF is observed during various infectious diseases including bursal disease virus [3, 4], Marek’s disease virus [5, 6], Fowl adenoviruses [7], *Escherichia coli,* fowl typhoid and *Salmonella* [8-10]. *Salmonella* enterica serovar Typhimurium (STm) is a gram-negative facultative anaerobic bacterium that causes serious clinical disease in newly hatched chicks [11], which is characterized by severe diarrhea, dehydration, and increased mortality [12]. STm infection results in immune suppression, cytokine imbalance and disruption of lymphoid tissue architecture [13]. The JNK signaling pathway plays a critical role during STm-induced thymic injury in mice [14, 15]. Although BF plays a significant role in STm infection in poultry [16], only few scientific reports have described the transcriptional changes in BF following STm infection in avian species.

Since the acquired immune system in the neonatal chicken is not completely functional until the first week, the innate immune system plays defensive crucial role for the chicks [17]. Bursa is important in the innate immunity of neonatal chicken and < 0.1% BF to body weight indicates infection in broilers [18, 19]. TLRs are key mediators of innate immune responses to various infections [20]. For gram negative bacteria, the endotoxin LPS is recognized by TLR2 and TLR4 [21, 22]. LPS stimulation disrupts the histo-morphological organization of different organs including BF of chicken [23, 24]. Reduced body and bursa weight are also characteristic of *salmonella* LPS stimulated chickens [25]. NF-κB and AP-1 are two key down-stream transcriptional factors of the TLR4 signaling pathways [26], which induce various inflammatory factors including TNF-α, IL-6, IL-8, IL-1b [26, 27]. Furthermore, chronic inflammation results in apoptosis of immune cells [28]. Inflammation in response to LPS results in induction of pro-apoptotic factors including nitric oxide, TNF-α and glucocorticoids [29, 30]. Although studies have described the immune response and atrophy of immune organs mediated by infectious diseases in model organisms like mice, rats as well as humans, the molecular mechanism of bursal atrophy is still largely unknown. Therefore, in the current study, we investigated the molecular mechanisms involved in acute bursal atrophy in young chickens induced by *Salmonella* LPS.

## RESULTS

### Bursal atrophy after LPS treatment

**Figure 1:**
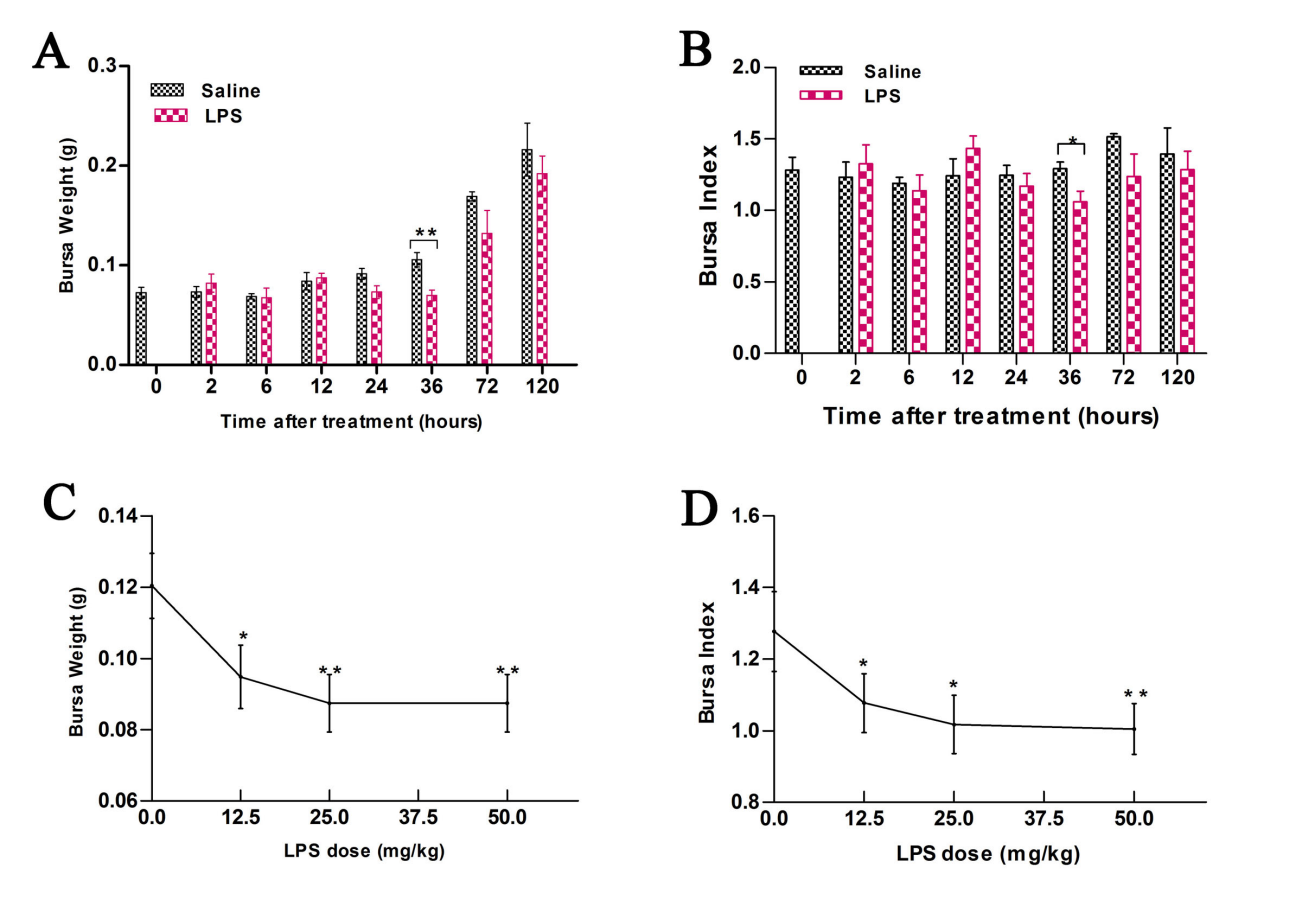
Bursal weight and index in chicks treated with LPS **A**. Comparison of weights of bursa of Fabricius (BF) in chicks at different time points (0-120 h) after treatment with 50 mg/kg LPS or saline. **B**. Comparison of bursal index in chicks at different time points (0-120 h) after treatment with 50 mg/kg LPS or saline. **C**. Comparison of weights of bursa of Fabricius (BF) in chicks at 36 h after treatment with different doses of LPS or saline. **D**. Comparison of bursal index in chicks at 36 h after treatment with different doses of LPS or saline. As shown, LPS induces acute bursal atrophy in chicks. Note: * denotes *P* < 0.05; ** denotes *P* < 0.01.

Bursal weight and bursal index were analyzed at 0, 2, 6, 12, 36, 72 and 120 h in chicks after intra-peritoneal injection of saline (control) or 50 mg/kg LPS (Figure 1). LPS treatment resulted in ∼ 36% decrease in bursal weight (*P* < 0.01, 0.07 ± 0.01 *vs*. 0.11± 0.02 g) and ∼ 18% in bursal index (*P* < 0.05, 1.06 ± 0.18 *vs*. 1.30± 0.11) at 36 h compared to control. At 72 h, LPS treatment resulted in ∼ 13% decrease in bursal weight (*P* > 0.05, 0.13 ± 0.03 *vs*. 0.15± 0.05 g) and ∼ 10% reduction in bursal index (*P* > 0.05, 1.24 ± 0.31 *vs*. 1.38± 0.21) compared to control. We also observed that the weight of bursa partially recovered to normal levels at 120 h post treatment (Figure 1A-1B). Furthermore, the decrease in bursal weight was associated with LPS dosage (Figure 1C-1D).

### Analysis of bursal morphology and ultrastructure after LPS treatment

**Figure 2:**
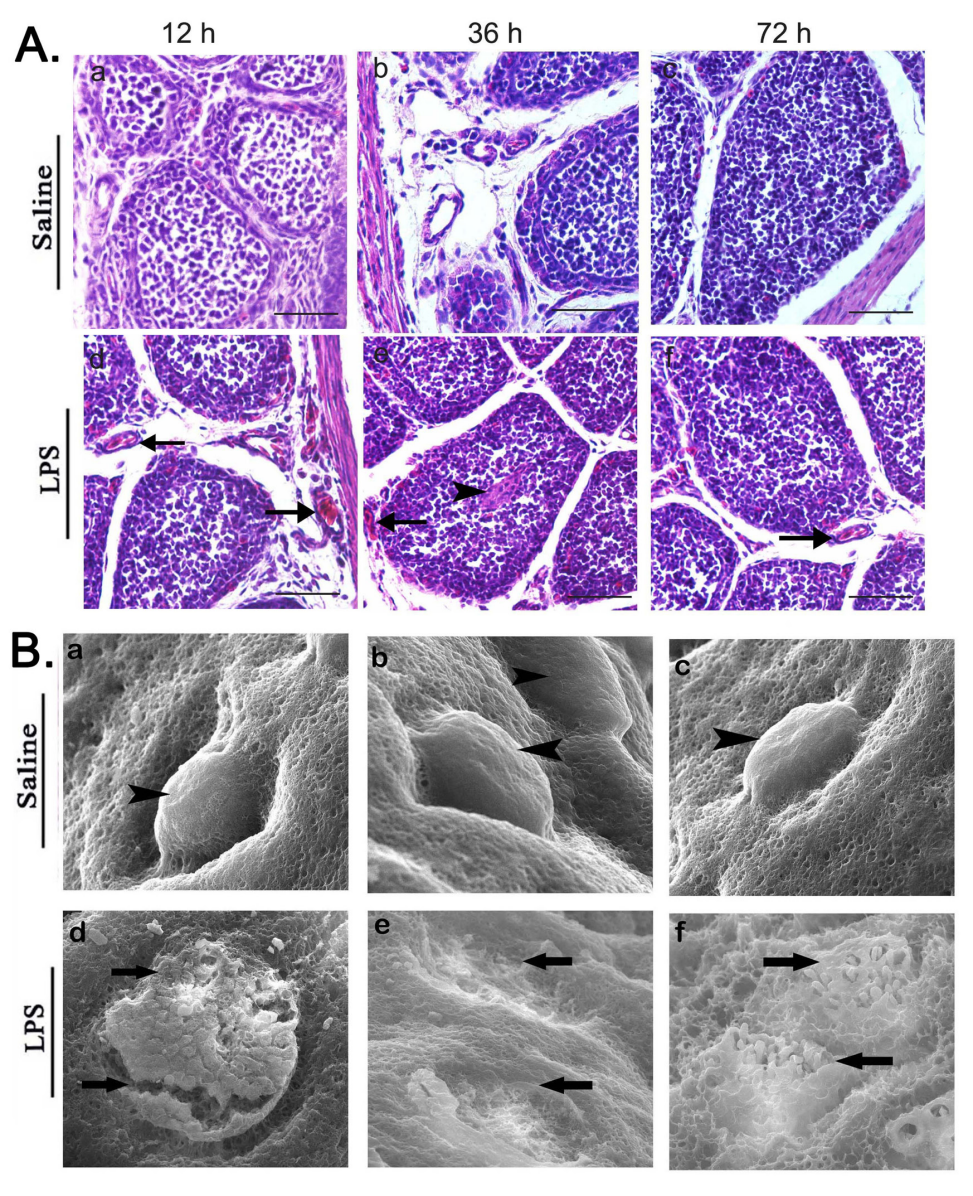
Analysis of bursal morphology after LPS treatment **A.** Representative images of H&E stained BF tissue sections at 12, 36 and 72 h after saline (control) or LPS i.p. injection. As shown, BF specimens from LPS-treated chicks show histopathological changes compared to the control. Arrows indicate blood vessel and arrow heads represent accumulation of RBC or inflammatory cells. Note: Scale bars = 50 μm. **B.** Representative scanning electron micrographs of BF sections at 12, 36 and 72 h after saline (control) or LPS i.p. injection showing the ultra structure of BF mucosal surface. Bursa tissue sections in LPS stimulated group show severe erosion and breaks in mucosal surface at 12 h, complete exuviation of bursal mucosal epithelial cells at 36 h and slight restoration of the disrupted mucosal surface at 72 h. Magnification = 500x. Arrowheads represent normal, smooth and intact bursal follicles on mucosal surface in saline group while arrows indicate broken, eroded and sloughed mucosal surface in LPS stimulated group. Data represent at least five tissue sections per chick per group (*n* = 3 at each time point).

H&E stained BF sections from LPS treated chicks showed infiltration of eosinophilic heterophils and increased number of nucleated oval shaped RBCs in or near blood vessels in chicken bursa at 12, 36 and 72 h time points (Figure 2A). The enhanced heterophilic granules accumulated in the medulla of bursal follicle and disrupted the outer boundary of bursal follicular cortex at 36 h post LPS stimulation (Figure 2A). Scanning electron micrographs showed distribution of disc-shaped polygonal epithelial cells on the mucosal surface of chicken bursa (Figure 2B). LPS stimulation also caused severe erosion and breaks of the mucosal membrane at 12 h and complete exuviation of bursal mucosal epithelial cells at 36 h. However, slight restoration of the disrupted mucosal surface was observed at 72 h (Figure 2B).

### Increased apoptosis and decreased proliferation in bursa after LPS treatment

**Figure 3:**
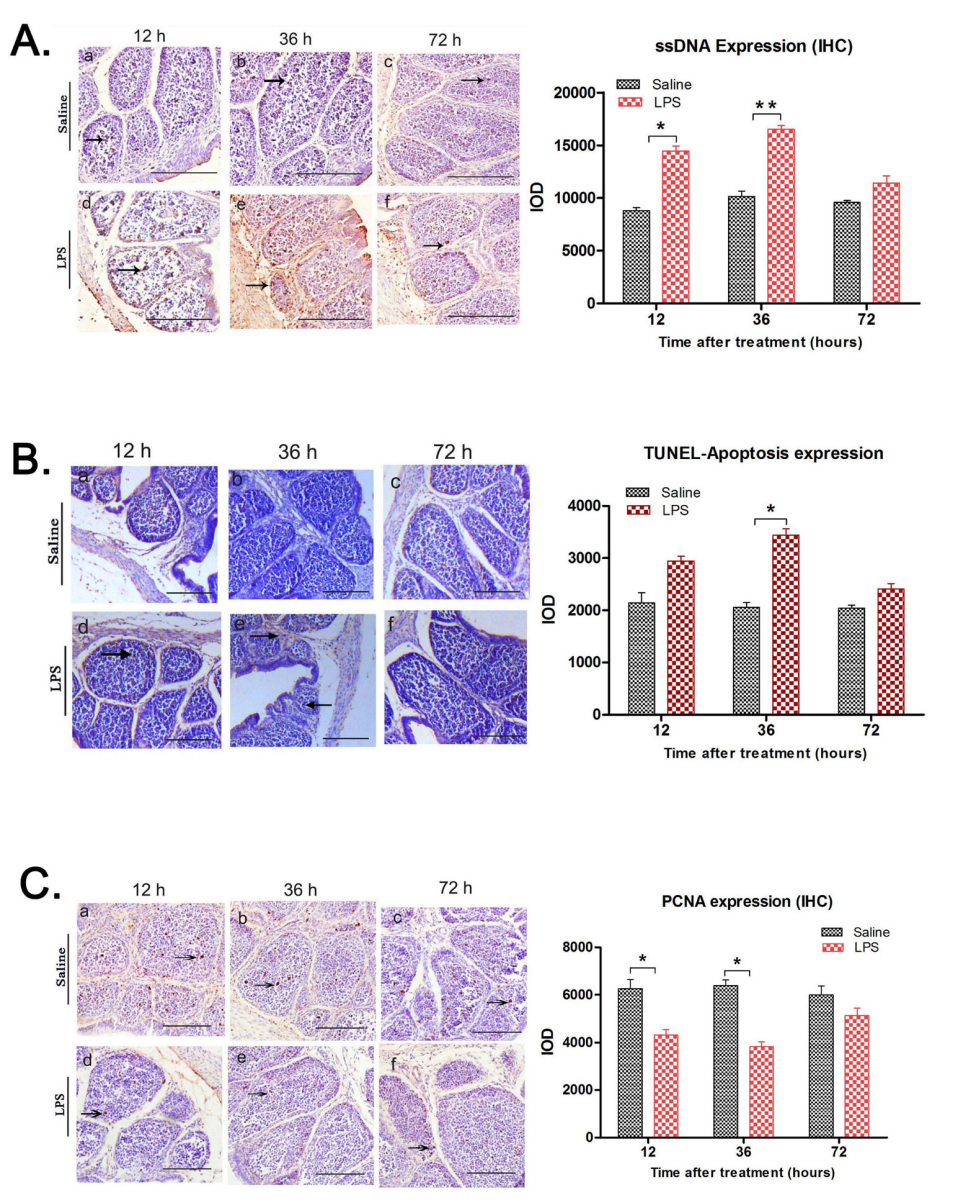
LPS treatment upregulates apoptosis and inhibits proliferation in chick BF **A.** Representative images of immunostaining with anti-ssDNA monoclonal antibody of BF tissue sections at 12, 36 and 72 h after saline (control) or LPS i.p. injection. Arrows show single stranded DNA (ssDNA) staining as a dark brown product (arrows). Integral optical density (IOD) analysis showed that in comparison to the saline group, ssDNA expression was upregulated in LPS treated group, especially at 12 (IOD ratio saline *vs* LPS; 8801:14453.67) and 36 h (IOD ratio saline *vs* LPS; 10135:16781.5). **B.** Representative images of TUNEL assay of BF tissue sections at 12, 36 and 72 h after saline (control) or LPS i.p. injection. Scale bars = 100 μm. IOD analysis shows increased TUNEL- positive (apoptotic) cells with brown stained nuclei in BF from LPS-treated chicks, especially at 36h time point (IOD ratio saline *vs* LPS; 2054.67:3439.67). **C.** Scale bars = 50μm. Serial tissue sections of chicken bursa of Fabricius were immuno-stained with PCNA antibody (proliferation marker) at 0h, 12 h, 36 h and 72 h post saline (control) or LPS stimulation **C.** Representative images of immunohistochemical staining of PCNA (proliferation) in BF tissue sections at 12, 36 and 72h after saline (control) or LPS i.p. injection. Arrows show PCNA positive signals as light brown dots. PCNA expression was downregulated in LPS treatment group compared to control. Integral optical density (IOD) of PCNA expression significantly decreased at 12 (IOD ratio saline *vs* LPS; 6266.67:4310) and 36 h (IOD ratio saline *vs* LPS; 6390:3822) after LPS treatment. Scale bars = 100μm; Note: * denotes *P <* 0.05; ** denotes *P* < 0.01. Data represent microscopic examination of at least five tissue sections per chick per group (*n* = 3 at each time point).

*In-situ* cell apoptosis in the bursa was analyzed by the formamide-MAb assay which recognizes damaged single-strand DNA (ssDNA) in early apoptotic cells. We observed increased ssDNA in bursa from LPS treatment group compared to the control (Figure 3A). Integral optical density (IOD) of ssDNA expression in LPS treatment group was increased at 12 (*P* < 0.05) and 36 h (*P* < 0.01) (Figure 3A). TUNEL assay showed that bursal sections from LPS treatment group had increased number of TUNEL-positive cells compared to control. High IOD value at 36h confirmed increased apoptotic cells in bursa from LPS-treated chicks compared to control (Figure 3B). Further, immunohistochemical analysis showed low PCNA expression in LPS group compared to the control group at 12 and 36 h (*P* < 0.05) suggesting decreased cell proliferation upon LPS treatment (Figure 3C).

### Identification of differential expressed genes (DEGs) in bursa after LPS stimulation

**Figure 4:**
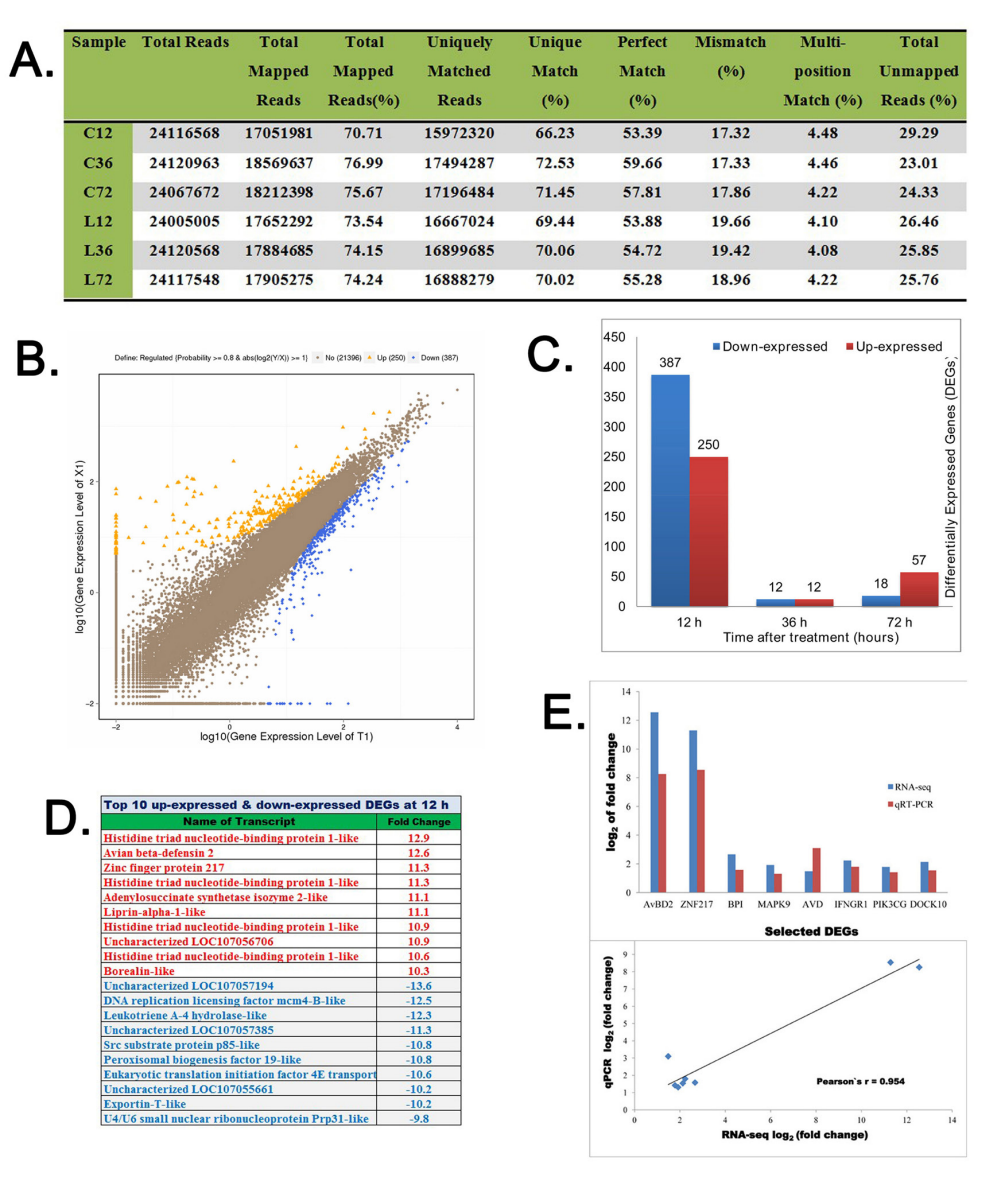
Analysis and validation of differentially expressed genes in LPS-treated chicken bursa **A.** Statistics of high-throughput sequencing reads aligned against reference genome. **B.** Scatter plot diagram showing log value of gene expression of LPS-treated BF samples (Y-axis) *versus* and log value of gene expression of saline treated BF samples (X-axis). The blue color indicates downregulated genes, orange color represents upregulated genes and brown color designates unchanged genes. Top legends on each figure show statistics of screening threshold values. **C.** The diagram shows total numbers of differential expressed genes (DEGs) at 12, 36 and 72h after saline or LPS treatments. **D.** Top 10 up-regulated and down-regulated DEGs at 12 h post saline or LPS treatments. The plus numbers designate upregulated log values while negative numbers represent downregulated log values of DEGs. **E.** Comparison and correlation analysis of log_2_ values of differential gene expression of eight genes (AvBD2, ZNF217, BPI, MAPK9, AVD, IFNGR1, PIK3CG and DOCK10) assessed by RNA-seq and qRT-PCR experiments.

The RNA-seq statistics pertaining to the average total reads, mapping reads and unique matching for each of the 18 samples are shown in Figure 4A. The differentially expressed genes (DEGs) between LPS and control (saline) group were analyzed by Random-Sampling Empirical Myerson (RSEM) software. Overall, there were 736 DEGs with 417 downregulated and 319 upregulated genes at all the three time points (12, 36 and 72 h; Figure 4B). Among these, we observed 637 DEGs (387 downregulated and 250 upregulated) at 12 h (Figure 4C), 24 DEGs (12 downregulated and 12 upregulated) at 36h and 75 DEGs (18 downregulated and 57 upregulated) at 72 h (Supplementary Table 1). Maximum DEGs were observed at 12 h time point. The top ten upregulated and downregulated DEGs at 12h are shown in Figure 4D. To validate the RNA-seq data, eight genes (AvBD2, ZNF217, BPI, MAPK9/JNK, AVD, IFNGR, PIK3CG, DOCK10) were randomly selected at 12h for qRT-PCR analysis, which confirmed the RNA-seq findings. Moreover, there was high correlation between qRT-PCR and RNA-seq data (*r* = 0.954; Figure 4E).

### Bioinformatics analysis of DEGs

**Figure 5:**
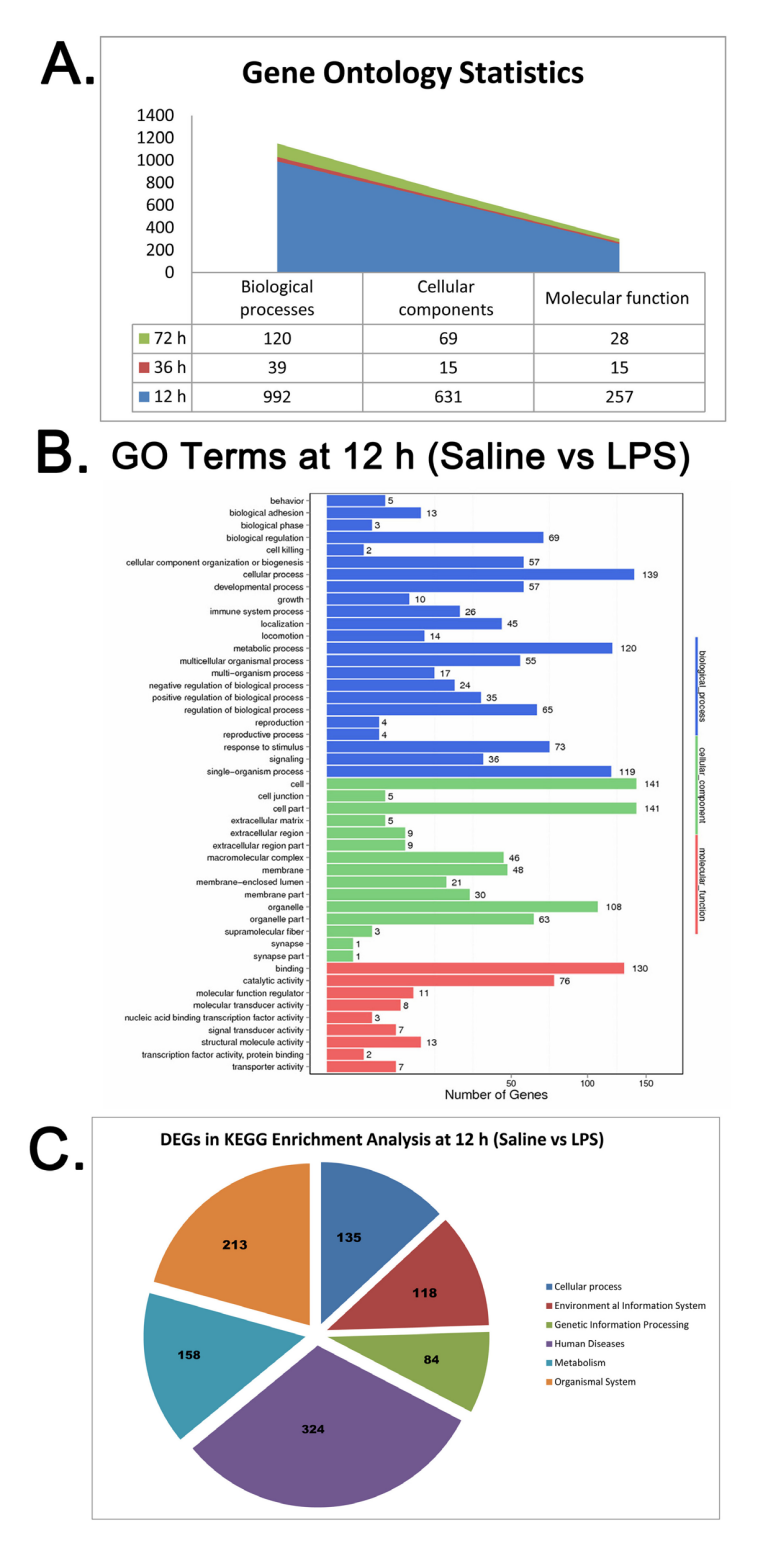
Gene Ontology (GO) and KEGG functional pathway analysis of DEGs in LPS-treated chicken bursa **A.** Gene Ontology (GO) statistical analysis in chicken bursa of Fabricius at 12, 36 and 72 h time points of RNA seq data from saline and LPS-treated chicken bursal samples. **B.** Diagram showing GO terms and their designated number of DEGs at 12 h post saline or LPS treatment chicken BF samples. X-axis shows number of DEG while Y-axis represents GO terms. Three different colors designate three ontologies of GO terms; blue color represents biological process, green color indicates cellular components and the red color shows molecular functions. **C.** Diagram showing number of DEGs involved in KEGG enrichment analysis at 12 h post saline or LPS treatment in chicken BF samples.

GO annotation of DEGs was performed at all the time points (12 h, 36 h and 72 h). All the DEGs were enriched in all three major GO classes, namely, biological process, cellular component and molecular function. Moreover, most DEGs were enriched in GO terms at 12 h. At 12 h, 992 genes were annotated in biological processes, 631 genes in cellular components and 257 genes in molecular function (Figure 5A). The top three enriched biological processes were cellular (139 genes), metabolic (120 genes) and single-organism (119 genes) processes (Figure 5B). The top three enriched cellular components were cell (141 genes), cell part (141 genes) and organelle part (108 genes) (Figure 5B). The top three enriched molecular function categories were binding (130 genes), catalytic activity (76 genes) and structural molecule activity (13 genes) (Figure 5B). KEGG enrichment analysis of DEGs at 12 h showed enrichment of 135 DEGs in cellular processes, 118 in environmental information system, 84 in genetic information processing, 324 human diseases, 158 metabolism and 213 organismal system related to the signaling pathways (Figure 5C). The details of these DEGs in each signaling pathway are listed in Supplementary Tables 1 and 2.

### TLR4-MAPK-NF-κB/AP-1 signaling pathway mediates acute bursal atrophy

**Figure 6:**
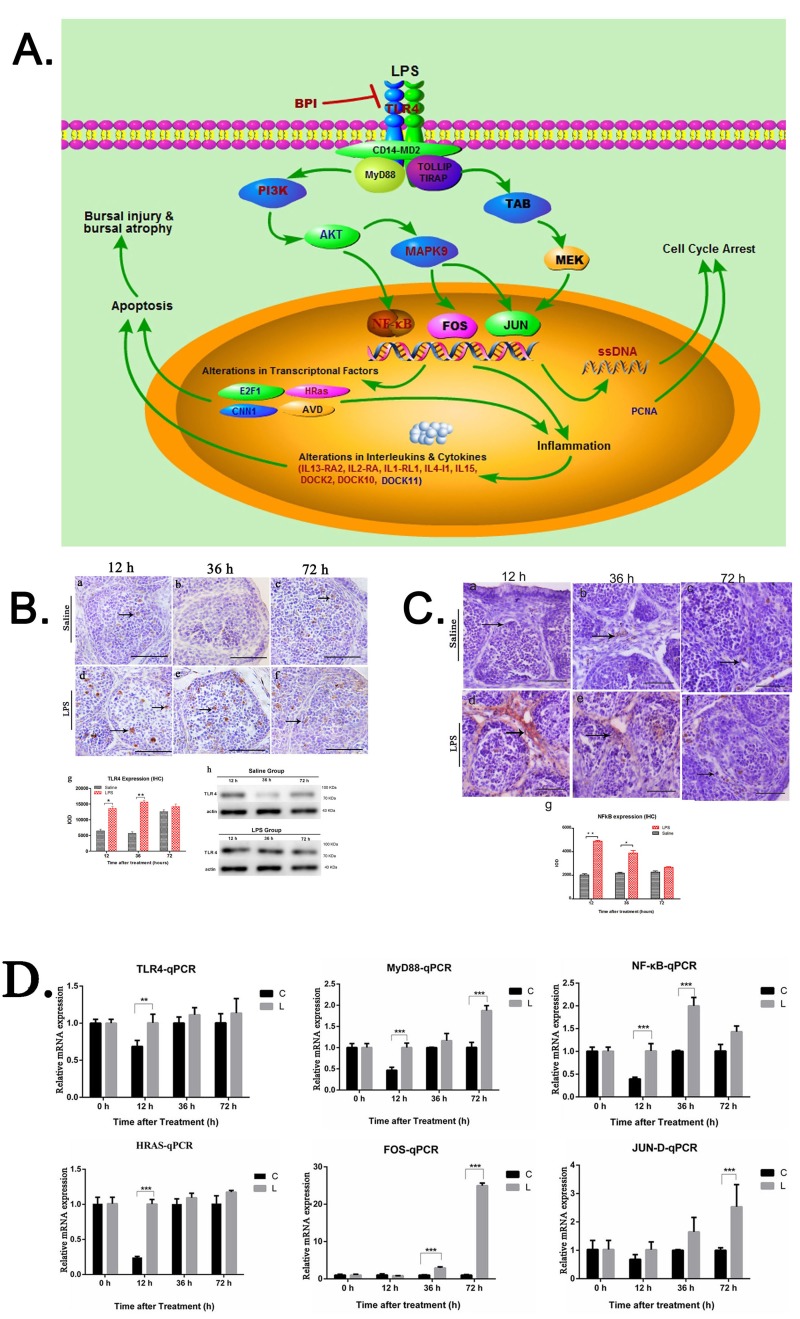
Functional pathway analysis and graphical illustration of molecular mechanism of LPS induced bursal atrophy **A.** Diagram showing TLR4-MAPK-NF-κB/AP-1 pathway analysis of differentially regulated genes in LPS-treated BF. The red colored text represents upregulated genes; blue designates downregulated genes and black designates neighboring genes in TLR4-MAPK-NF-κB/AP-1 pathway in chicken bursa of Fabricius at early time point. The figure was drawn in Pathway Builder Tool. 2 software. **B.** Representative images of immunostained BF sections from LPS and saline control group chicks using anti-TLR4 antibody at 12, 36 and 72 h time points. As seen, TLR4 expression is enhanced in the LPS treated BF compared to control. Integral optical density (IOD) shows increased TLR4 expression at 12 and 36 h after LPS treatment. Scale bars = 50μm. Note: * denotes *P* < 0.05; ** denotes *P* < 0.01. Data represent microscopic examination of at least five tissue sections per chick per group (*n* = 3 at each time point). Also shown are immunoblots showing increased TLR4 expression in LPS-treated BF compared to control. **C.** Representative images of immunostained BF sections from LPS and saline control group chicks using anti-NFkB-p50 antibody at 12, 36 and 72 h time points. IOD analysis shows increased NFkB expression at all 3 time points in LPS-treated BF (arrows) compared to control. Scale bars = 50μm. * denotes *P* < 0.05; ** denotes *P* < 0.01. Data represent microscopic examination of at least five tissue sections per chick per group (*n* = 3 at each time point). **D.** QRT-PCR analysis of TLR4, MyD88, NFκB, HRas, FOS and JUN-D mRNA expression at 12, 36 and 72 h post LPS treatment as validation of key components of TLR4-MAPK-NF-κB/AP-1 pathway.

Integrated pathway analysis was performed for the DEGs in the KEGG database (http://www.kegg.jp/kegg/tool/map_pathway2.html). A number of DEGs were involved in TLR4-MAPK signaling pathway including TLR4, BPI, PI3K, AKT, MAPK9 genes. Also, inflammatory factors such as IL13-RA2, IL2-RA, IL1-RL1, IL4-I1, IL15 DOCK2, DOCK10 and DOCK11 were differentially expressed. These inflammatory factors also induce apoptosis. Therefore, we identified TLR4-MAPK-NF-κB/AP-1 as the key signaling pathway mediating acute bursal atrophy during LPS treatment (Figure 6A). Moreover, we validated the expression of the key genes involved in this signaling pathway. TLR4 was upregulated and constitutively localized in the cortico-medullary regions and follicular medulla at 12 and 36h (*P* < 0.05; Figure 6B). Similarly, NF-κB was upregulated at 12 and 36h (*P* < 0.05; Figure 6C). Moreover, qRT-PCR results showed that TLR4, MyD88, NF-κB, HRas and sub-units of AP-1 (FOS and Jun-D) were significantly up-regulated 12, 36 and 72 h after LPS treatment (Figure 6D).

## DISCUSSION

Acute atrophy of bursa is observed in various infectious diseases including *Salmonella* infection. In the present study, we found that LPS stimulation markedly reduced bursal weight and index. Also, the structural integrity of bursa was disrupted due to inflammation. We also identified that TLR4-MAPK-NF-κB/AP-1 signaling pathway played an important role in acute atrophy of bursa induced by LPS.

In this study, we observed morphological manifestation of atrophy at 36 h after LPS treatment. However, in regards to gene expression changes, the most significant time point was 12 h. Therefore, the molecular response to the LPS stimulation was earlier than the appearance of the morphological atrophy phenotype. In our previous study, we found thymus atrophy at 36 h and gene expression changes at 12 h after LPS treatment [14, 15]. Therefore, both bursa and thymus show similar phenotype changes in response to LPS treatment. In addition, there were no significant changes in bursal weight between LPS treatment and control group at 72 h and 120 h suggesting compensatory growth after atrophy in the LPS treatment group. SEM results showed that the structure of bursa was disrupted at 12 h and damaged considerably at 36 h after LPS treatment. However, it recovered at 72 h, but was not completely restored at 120 h. In a previous study, reduction in feeding efficiency and growth performance along with compensatory growth mechanism was observed in broiler chicks following LPS challenge [31]. Therefore, although the weight of bursa was similar to the control group at 72 h, its function was not fully restored.

We also observed increased apoptosis and decreased proliferation at 12 and 36 h after LPS treatment, which resulted in the bursal atrophy. Many studies have confirmed that inflammation induces cellular apoptosis [32-34]. In our previous study in chicken, inflammation induced apoptosis in thymus at 12, 36 and 72 h after LPS treatment [14, 15]. In the present study, we observed infiltration of eosinophilic heterophils and nucleated oval shaped RBCs in or near blood vessels in chicken bursa at 12, 36 and 72 h time points. Enhanced heterophilic granules accumulated in the medulla of bursal follicle at 36 h post LPS stimulation. Moreover, RNA-seq results showed upregulation of a number of inflammatory factors after LPS treatment. All these results indicated that inflammation due to LPS resulted in cellular apoptosis lead to bursal atrophy.

The RNA-seq data showed that many DEGs were significantly enriched in immune response pathways. Also, integrated pathway analysis revealed that TLR4-MAPK-NF-κB/AP-1 was the major pathway responsible for atrophy of bursa induced by LPS. The expression of major genes in this signaling pathway, including TLR4, MAPK9, NF-κB and Fos/Jun (two subunits of AP-1) were significantly up-regulated after LPS treatment. Previous studies have confirmed that TLR4 is the receptor of LPS [35-37]. TLR4 is required for inflammatory responses after intravenous LPS injection [38]. MAPK9 is induced and activated by TLR4 with mediation by PI3K/AKT phosphorylated kinases [39, 40].

Moreover, NF-κB and AP-1 are induced and activated by MAPK and MEK [41, 42]. NFκB and AP-1 are two key transcriptional factors for inflammation, which induce expression of inflammatory factors including IL-1, IL-6 and TNF-α [43, 44]. Also, c-Fos and c-Jun transcriptional factors are regulated by NF-κB and AP-1 [45, 46]. Moreover, these transcriptional factors participated in cellular apoptosis and inflammation [47]. Therefore, our findings show that the TLR4-MAPK-NF-κB/AP-1 pathway is the major signaling pathway mediating inflammation and bursal atrophy after LPS treatment in chicken.

In conclusion, our study demonstrates that LPS treatment induces TLR4-MAPK-NF-κB/AP-1 signaling pathway resulting in increased inflammation and apoptosis in the bursa, thereby leading to its atrophy.

## MATERIALS AND METHODS

### Ethics statement

The present study was approved by the Animal Care and Use Committee of Huazhong Agricultural University (HZAU), Wuhan, China.

### Experimental design and lipopolysaccharide (LPS) stimulation

Healthy one-day-old Cobb strain broiler chicks were obtained from a local breeding company (Zhengda, Wuhan, China). The chicks were reared under a conventional housing environment similar to commercial broiler husbandry conditions. Water and feed were provided *ad libitum* without any medication and vaccination. In a separate study, the effect of different doses of LPS (i.e., 12.5, 25 and 50 mg/kg b.w.) on bursa weight was determined. In the present study, the chicks were intraperitoneally (i.p.) injected with 50mg/kg *salmonella* LPS (L7261; Sigma-Aldrich, St. Louis, MO, USA) in 0.5ml saline (0.75 % NaCl solution); control chicks received only 0.5ml saline i.p.

### Bursal tissue harvesting

The chicks were sacrificed at various time points and the BF were harvested and weighed. The bursal index (BI) was calculated as

Bursal index = BF weight (g) /body weight (g) x 1000

Six tissue samples were collected for each experimental treatment group (LPS or saline) at 0, 2, 6, 12, 36, 72 and 120 hours post saline and LPS treatments. Tissues were snap frozen in liquid nitrogen and then preserved at -70 °C for RNA-seq and other experiments. Tissue samples were also fixed in 2.5% glutaraldehyde solution at 4°C for scanning electron microscopy (SEM). For histological examination, tissue samples were fixed in 4% paraformaldehyde at room temperature, dehydrated and then embedded in paraffin wax. Then, 5μm serial tissue sections were cut using a Leica microtome (Nussloch Gmbh, Germany) and mounted on polylysine-coated slides (Boster Corporation, China). The tissue sections were dried at 37°C overnight.

### Hematoxylin and eosin (H&E) staining

Briefly, bursal specimens were deparaffinized in xylene and rehydrated in a graded series of decreasing ethanol concentrations. The sections were stained in Harris hematoxylin solution (Baso, China) for 4 min, counterstained in eosin solution, dehydrated with ascending concentrations of ethanol, cleared in xylene, and fixed by mounting a cover slip using neutral balsam. Stained tissue sections were examined by light microscopy (Olympus BX51, Tokyo, Japan) with a digital camera (DP72; Olympus).

### Scanning electron microscopy (SEM)

Scanning electron microscopy (SEM) was performed as previously described [48]. Briefly, BF tissues were post-fixed in osmium tetroxide (OsO_4_), then washed in 0.1M cacodylate buffer and dehydrated in ascending acetone concentrations. Bursal tissues were then impregnated with pure hexamethyldisilazane (HMDS) (Sigma-Aldrich, St. Louis, USA) and air dried. After drying, the samples were mounted on aluminum stubs, coated with a layer of gold sputter coater and viewed in a scanning electron microscope (Hitachi SU8010).

### Immunohistochemical staining

Immunohistochemical staining of BF sections was carried out using antibodies against proliferative cell nuclear antigen (PCNA) (1:200), TLR4 (1:100) and NF-κB-p50 (1:250) (Santa Cruz Biotechnology, Inc., Santa Cruz, CA, USA) following previously described methods [49]. Formamide-monoclonal antibody (MAb) assay was performed with the anti-ssDNA monoclonal antibody (1:20; EMD Millipore, Billerica, USA) as previously described [14, 15]. This assay differs from the traditional immuno-staining protocol by having an additional pre-treatment of bursal tissue sections with saponin (0.1mg/ml) and proteinase K (20μg/ml) in PBS at 37°C for 20 min, followed by incubation at 56°C for 20 min in formamide diluted 50% (v/v) with dd-H_2_O. Also, unlike the routinely used heat induced antigen retrieval in a micro oven, here the sections were treated with ice cold PBS for 5 min and then incubated with anti-mouse IgM SABC kit (Boster, Wuhan, China) in place of another standard secondary antibody kit. Hematoxylin was used for counterstaining.

### Analysis of bursal apoptosis by TUNEL Assay

TUNEL staining was carried out with the *In Situ* Cell Death Detection Kit-POD (Roche, Mannheim, Germany) according to previously described method [50]. Briefly, 5μm serial bursal tissue sections were deparaffinized in xylene and then rehydrated in descending concentrations of ethanol. Endogenous peroxidase activity was blocked with 3% H_2_O_2_ for 10 min followed by Proteinase K treatment for 20 min. Then, the specimens were incubated with 1:20 diluted terminal deoxynucleotidyl transferase (TdT) in a reaction buffer (with fixed concentrations of digoxigenin-labeled nucleotides) for 90 min at 37-40°C in humidified chamber. After washing slides with Stop/Wash buffer, the tissue sections were again incubated with the pre-diluted anti-digoxin antibody (1:100) for 60 min at 37-40°C in humidified chamber. Tissue sections were then incubated with the 3, 3′- diaminobenzidine (DAB) chromogen (Boster, Wuhan, China) and counterstained with hematoxylin.

### Semi-quantitative analysis of protein expression in bursal tissue sections

A light microscope (BH-2; Olympus, Japan) with an attached digital camera (DP72; Olympus) was used for the examination of serial bursal tissue sections. The expression of positive signal was assessed under high-power fields at random. Same microscope with attached camera set was used for all the micrographs. Integral optical density (IOD) for positive staining was calculated using Image-Pro Plus (IPP) 6.0 software (Media Cybernetics, USA). GraphPad Prism software version 5.0 (GraphPad Software, Inc., San Diego, USA) was used for graphing the data.

### Western blot analysis

Western blotting was performed following previously described methods [51]. Briefly, the frozen specimens were powdered in liquid nitrogen and homogenized in lysis buffer with a protease inhibitor enzyme. The supernatants were vortexed, incubated on ice and centrifuged at 12,000 × *g* for 5 min. The protein concentrations was measured using BCA protein quantification kit (Vazyme™ biotyech.co., ltd.). Equal amounts of total proteins (40 μg) were subjected to 10% SDS-PAGE (30 min at 80 volts and after that 60 min at 100 volts). Then, the separated proteins were transferred onto a PVDF membrane (Merck Millipore, USA). The membranes were incubated against rabbit anti-TLR4 (A00017-Boster, 1:400) and anti-human β-actin (sc-47778-Santa Cruz, 1:1000) antibodies for 12 h and after washing in 1X TBST buffer thrice, were incubated with peroxidase conjugated secondary antibody (1:3000) for 30 min (Boster, China). The blots were developed with Super Signal West Pico Chemiluminescent Substrate (Thermo Fisher Scientific, Waltham, MA, USA) and visualized using ChemiDoc-It™ Imaging System.

### Quantitative real-time PCR (qRT-PCR)

Total RNA from bursal tissue samples was isolated using Trizol (Invitrogen, Carlsbad, CA, USA) following the manufacturer`s instructions. The genomic DNA (gDNA) was removed by treating RNA with RNase-free DNase I (Fermentas, Opelstrasse, Germany). RevertAid First Strand cDNA Synthesis Kit (Fermentas, Opelstrasse, Germany) was used to synthesize of first strand cDNA. For qPCR, 10μl total reaction mixture was comprised of 5μl SYBR Select Master Mix for CFX (Applied Biosystems), 2μl of each forward and reverse primer and 1μl of template cDNA. The qPCR reactions were performed on a Bio-Rad CFX Connect real-time PCR detection system (Bio-Rad, Hercules, CA, USA). The qPCR conditions were as follows: pre-denaturation at 95 °C for 5 min, followed by 40 cycles of denaturation at 95 °C for 30 s, annealing at 60 °C for 30 s, and elongation at 72 °C for 20 s. All samples were run in triplicate and relative gene expression levels were quantified using the ΔΔCt method using β-actin as control. Primers sequences for qPCR are given in Table 3.

**Table 1:** Differentially expressed genes (DEGs) and related KEGG Pathways

DEGs associated with immunity and signal transduction pathways (at 12h post saline/LPS treatment)				
No. of DEGs	Upregulated genes	Downregulated genes	Pathway	P-value
10	PIK3CA, LOC107056430, LCP2, MAPK9, PIK3CG	LOC107050724, LOC107056737, TAGLN, LOC107057160, LOC101750560	Fc epsilon RI signaling pathway (ko04664)	0.003053823
12	PIK3CA, IL15, CCLI10, MAPK9, RPS6KA5, PIK3CG	RLTPR, CNN1, LOC107057160, LOC101750560, LOC107050645, TAGLN,	TNF signaling pathway (ko04668)	0.00772329
15	PIK3CA, LOC107056430, CCLI10, CCR2, DOCK10, DOCK2, PIK3CG, CXCL14, CXCL12	LOC107050724, SASH3, PYGO2, LOC101750560, PDLIM7, TAGLN	Chemokine signaling pathway (ko04062)	0.008371477
24	NID1, F13A1, PIK3CA, MMP2, FGL1, CXCL12, LOC107056639, ITGAV, SPECC1L, CDH5, PIK3CG	TESPA1, ACTN4, TAGLN, MYL9, PDLIM7, LOC107057430, LOC107050460, LOC107050724, LOC101750377, LOC101747592, LOC107056858, LOC107050373, LOC107051991	Leukocyte transendothelial migration (ko04670)	0.00961218
9	PIK3CA, LOC107056430, PAK3, MAPK9, PIK3CG	SASH3, PYGO2, LOC107057160, LOC101750560	ErbB signaling pathway (ko04012)	0.02833687
10	PIK3CA, LOC107056737, DOCK10, PIK3CG	LOC107050724, SASH3, CFL2, LOC101750560, TAGLN, DOCK2	Fc gamma R-mediated phagocytosis (ko04666)	0.03687679
9	BPI, TLR2A, PIK3CA, CD28, CCLI10, MAPK9, PIK3CG, LOC107057160	LOC107057160, LOC101750560	Toll-like receptor signaling pathway (ko04620)	0.05084102
18	IL1R2, LOC107056430, PDGFRA, FGF7, MAPK9, TGFBR1, RPS6KA5	HSPB1, SASH3, CNN1, JUN-D, LOC107057160, LOC101750560, RPS6KA3, CDC25B, LOC107051014, TAGLN, FLNA	MAPK signaling pathway (ko04010)	0.06491393

DEGs associated with cancer pathways (at 12h post saline/LPS treatment)				
26	PIK3CA, DICER1, MMP2, FGL1, FGF7, GOLIM4, LOC107056430, PDGFRA, ZEB2, HNRPK, RPS6KA5, PIK3CG, LOC107056494	LOC107057430, LOC107050460, LOC101750377, SASH3, E2F1, LOC107056068, PYGO2, TPM1, LOC101747592, LOC107050373, TESPA1, RASSF1, CDC25B	MicroRNAs in cancer (ko05206)	0.00172622
8	LOC107056430, FGF7, RPS6KA5, LOC107055989, MMP2, LOC107056858	E2F1, RASSF1	Bladder cancer (ko05219)	0.008974338
30	PIK3CA, MMP2, FGL1, LOC107056430, FGF7, LOC107055989, FAM46C, LOC107056858, NID1, TLR2A, F13A1, PIK3CG, NUDT16L1, DCN, ITPR2, LUM, LOC107056639, ITGAV	TESPA1, PDLIM7, FLNA, TAGLN, LOC107056482, LOC107057430, LOC107050460, LOC101750377, LOC101747592, LOC107050373, LOC101750560, LOC107050724	Proteoglycans in cancer (ko05205)	0.01710184
11	PIK3CA, LOC107056430, FGF7, PIK3CG, PDGFRA, GSTA3	E2F1, LOC101750560, CDK2, LOC107050491, LOC107056196	Prostate cancer (ko05215)	0.02190454
7	PIK3CA, PIK3CG, TGFBR1, MAPK9, JAK1	E2F1, LOC101750560	Pancreatic cancer (ko05212)	0.02477662
8	PIK3CA, LOC107056430, PDGFRA, PIK3CG, LOC107056858, FGF7	E2F1, LOC101750560	Melanoma (ko05218)	0.02656947
8	PIK3CA, LOC107056430, PIK3CG, PDGFRA	PYGO2, E2F1, LOC101750560, CABP2	Glioma (ko05214)	0.03668416
8	PIK3CA, LOC107056430, PIK3CG, TGFBR1	SASH3, PYGO2, E2F1, LOC101750560	Chronic myeloid leukemia (ko05220)	0.03810799

DEGs associated with metabolic pathways (at 12h post saline/LPS treatment)				
14	ATP5A1W, ATP12A	ATP5D, LOC107056507, SDHC, LOC107049545, NDUFA13, ATP5G2, NDUFA2, Dock11, LOC107049047, COX5A	Oxidative phosphorylation (ko00190)	0.00017938
5	ALDH1A3, IL4I1,	LOC107056850, MIF, LOC107049269	Phenylalanine metabolism (ko00360)	0.001328849
4	-----------	LSS, SC5D, LOC100857622, LOC107056717	Steroid biosynthesis (ko00100)	0.009269242
4	-----------	CHURC1, LOC107055323, FDPS, LOC107049309	Terpenoid backbone biosynthesis (ko00900)	0.01784414
4	GSTA3, ALDH1A3	LOC107050491, LOC107056196	Drug metabolism - cytochrome P450 (ko00982)	0.04348163

DEGs associated with all significant pathways(at 36h post saline/LPS treatment)				
3	LOC107049120, YF6, LOC768350	-----------	Graft-versus-host disease (ko05332)	0.000271798
3	LOC107049120, YF6, LOC768350	-----------	Allograft rejection (ko05330)	0.000503261
5	LOC107049120, YF6, LOC768350, BG2	THBS1	Phagosome (ko04145)	0.000544606
3	LOC107049120, YF6, LOC768350	-----------	Antigen processing and presentation (ko04612)	0.000688693
3	LOC107049120, YF6, LOC768350	-----------	Primary immunodeficiency (ko05340)	0.000730337
3	LOC107049120, YF6, LOC768350	-----------	Autoimmune thyroid disease (ko05320)	0.001049398
3	LOC107049120, YF6, LOC768350	-----------	Natural killer cell mediated cytotoxicity (ko04650)	0.00364005
3	LOC107049120, YF6, LOC768350	-----------	T cell receptor signaling pathway (ko04660)	0.00433848
4	LOC107049120, YF6, LOC768350, BG2		Endocytosis (ko04144)	0.004680683
3	LOC107049120, YF6, LOC768350	-----------	Hematopoietic cell lineage (ko04640)	0.007984066
3	LOC107049120, YF6, LOC768350	-----------	Herpes simplex infection (ko05168)	0.008757677
4	LOC107049120, YF6, LOC768350	THBS1	Cell adhesion molecules (ko04514)	0.009543652
3	LOC107049120, YF6, LOC768350	-----------	Viral carcinogenesis (ko05203)	0.01212585
3	LOC107049120, YF6, LOC768350	-----------	Epstein-Barr virus infection (ko05169)	0.01801051

DEGs associated with all significant pathways (at 72h post saline/LPS treatment)				
4	-----------	LOC769899, AVD, AVR2, LOC768350	Graft-versus-host disease (ko05332)	0.000462069
4	-----------	LOC769899, AVD, AVR2, LOC768350	Allograft rejection (ko05330)	0.001016396
6	CTGF, CYR61	LOC769899, AVD, AVR2, LOC768350	Hematopoietic cell lineage (ko04640)	0.001108016
4	-----------	LOC769899, AVD, AVR2, LOC768350	Antigen processing and presentation (ko04612)	0.001513009
4	-----------	LOC769899, AVD, AVR2, LOC768350	Primary immunodeficiency (ko05340)	0.001629486
4	LOC100857334, LOC770025, LOC107049046, 107055485	-----------	Ribosome (ko03010)	0.001710519
6	-----------	LOC769899, AVD, AVR2, LOC768350, LOC107055757, HNRPK	Viral carcinogenesis (ko05203)	0.002413858
5	FOS	LOC769899, AVD, AVR2, LOC768350	T cell receptor signaling pathway (ko04660)	0.002489395
4	-----------	LOC769899, AVD, AVR2, LOC768350	Autoimmune thyroid disease (ko05320)	0.002569968
7	CTGF, CYR61, LOC100857858	LOC769899, AVD, AVR2, LOC768350	Phagosome (ko04145)	0.003478134
4	-----------	LOC769899, AVD, AVR2, LOC768350	Natural killer cell mediated cytotoxicity (ko04650)	0.01183826
2	-----------	LOC107052718, LOC107052719	Folate biosynthesis (ko00790)	0.01851986
5	CTGF, FOS, LOC107049866, LOC107049603, CYR61	-----------	Osteoclast differentiation (ko04380)	0.03108378
6	CTGF, CYR61	LOC769899, AVD, AVR2, LOC768350	Cell adhesion molecules (ko04514)	0.03301481
2	FOS, LOC769000	-----------	Colorectal cancer (ko05210)	0.03620476
5	LOC769000	LOC769899, AVD, LOC768350, AVR2	Endocytosis (ko04144)	0.04666407

**Table 2:** KEGG orthology and GO Terms associated with selected differentially expressed genes (DEGs)

DEGs	Aliases	Gene product	Log value	GO term/s	KEGG Orthology
BPI	LBP, BPIFD2	bactericidal/permeability-increasing protein	2.666167	------	K05399; lipopolysaccharide-binding protein
PIK3CG	PI3CG, PI3K, p120-PI3K	phosphatidylinositol-4,5-bisphosphate 3-kinase, catalytic subunit gamma	1.784928	------	K00922; phosphatidylinositol-4,5-bisphosphate 3-kinase
TLR2A	TLR2, CD282, TIL4	toll-like receptor 2 family member A	1.9210023	GO:0038023, GO:0031224, GO:0006954, GO:0002224, GO:0001817, GO:0009607	K04440;c-Jun N-terminal kinase
MAPK9	JNK/ JNK2-α1	mitogen-activated protein kinase 9	1.9210991	------	K04440; c-Jun N-terminal kinase
IFNGR1	IMD27A, IMD27B, CD119	interferon gamma receptor 1	2.2324337	GO:0005515	K05132; interferon gamma receptor 1
USp53	p53, TP53, BCC7, TRP53	ubiquitin specific peptidase 53	4.4560819	------	K14206; solute carrier family 15 (oligopeptide transporter), member 1
JUN-D	AP-1, Jun-D	jun D proto-oncogene	-1.5720083	------	K04449; transcription factor jun-D, K04440; c-Jun N-terminal kinase
SASH3	CRK, CRKII, p38	SAM and SH3 domain containing 3	-1.5287857	------	K04438; proto-oncogene C-crk
HSP27	HSPB1	heat shock protein family B (small) member 1	-3.2252416	------	K04455; heat shock protein beta-1
AVD	MHC class 1	Avidin	1.48434927	------	K06458; CD8A, K06459; CD8B, K06751; major histocompatibility complex, class I, K15214; TATA box-binding protein-associated factor RNA polymerase I-C
E2F	RBP3	E2F transcription factor 1	-1.3682659	GO:0005515, GO:0001071, GO:0044212, GO:0005515, GO:0001071, GO:0044212, GO:1901990, GO:0006974, GO:0012501, GO:0043488, GO:0008284, GO:0000083, GO:1901990, GO:0006974, GO:0012501, GO:0043488, GO:0008284, GO:0007420, GO:0000083	K17454; transcription factor E2F1
CNN1	HEL-S-14, SMCC, Sm-Calp	calponin 1, basic, smooth muscle	-3.3097164	GO:0043232, GO:0008092, GO:0008092, GO:0030036	K15411, paired immunoglobulin-like type 2 receptor alpha, K04426; mitogen-activated protein kinase 5, K06712; butyrophilin, K06084; F-box protein 20
LOC101750560	Akt, akt1, PKB	uncharacterized LOC101750560	-1.6138883	------	K04456, RAC serine/threonine-protein kinase
LOC107056430	HRas, GTPas Ras, Ras	GTPase HRas	3.0362118	GO:0012505, GO:0043231, GO:0044444, GO:0016020, GO:0017111, GO:0032550, GO:0005515, GO:0000075, GO:0043405, GO:0031532, GO:0031346, GO:0043410, GO:0009653, GO:0010586, GO:0007569, GO:0016477, GO:0012502, GO:0007254, GO:0016601, GO:0006935, GO:0007599	K02833; GTPase HRas

**Table 3:** QRT-PCR primer list

Gene Symbol	Genbank Accession Number	Oligonucleotide sequence(5'to3')	
		Forward	reverse
AvBD2	NM_001201399.1	TTCTCCAGGGTTGTCTTCGC	TGCATTCCAAGGCCATTTGC
ZNF217	XM_015296520.1	CCACTGATGTACGAGAGTGCT	TACAAAAATGGGGCAGGTGCT
BPI	XM_417465.5	ATCTGGCCACATCGTAGGGA	CGTCAGCTTTATCTGCGCTC
MAPK9	NM_205095.1	ATGAGTGACAGTAAATGCGA	TCGCATTTACTGTCACTCAT
PIK3CG	XM_015275714.1	ATGGAGTTGGGTGACTATGA	TCATAGTCACCCAACTCCAT
DOCK10	XM_015277024.1	ATGGGTTGTGCTGCCAGCAT	ATGCTGGCAGCACAACCCAT
IFNGR1	NM_001130387.1	ATGGCCGAGCGCCGCGTGCC	GGCACGCGGCGCTCGGCCAT
AVD	NM_205320.1	ACACCATCAACAAGAGGACCC	AAGATGTTGATGCCGACCCT
TLR4	NM_001030693.1	TGAAAGAGCTGGTGGAACCC	CCAGGACCGAGCAATGTCAA
MyD88	NM_001030962.2	AGGATGGTGGTCGTCATTTC	TTGGTGCAAGGATTGGTGTA
NF-κB	NM_001030962.2	CTACTGATTGCTGCTGGAGTTG	CTGCTATGTGAAGAGGCGTTGT
HRAS	NM_205292.1	TAGAAACGTCGGCCAAAACC	CCACTCTCATCTGGTGGGTT
FOS	NM_205508.1	CCCGTCAACTCGCAGGATTT	CGGCGGATCCTCCTCTTTTC
Jun-D	XM_015300148.1	CCCATCGATATGGACACGCA	TCCTGAGCGGCGTTTTTACT
β-actin	NM_205518.1	TTGTTGACAATGGCTCCGGT	TCTGGGCTTCATCACCAACG

### RNA-seq and data analysis

TRIzol (Invitrogen, Carlsbad, CA, USA) was used to isolate total RNA from BF samples and the quality of RNA was assessed using Nanodrop spectrophotometer (Thermo, Waltham, MA, USA) and by gel electrophoresis. For high-throughput sequencing (RNA-seq), 18 BF RNA samples (three time points at 12, 36 and 72 h; three biological replicates at each time point and two treatments,namely, saline and LPS) were selected in equal quantities. Briefly, after construction of strand-specific cDNA libraries, sequencing was performed on Illumina HiSeq 4000 platform by BGI Co., Ltd. (Shenzhen, China). The original raw data was saved in FASTQ/FQ file format. Bowtie2 was used for mapping of clean reads to reference gene and HISAT to reference chicken genome (release: *Gallus gallus* 5.0). The gene expression was quantified by FPKM (fragments per kilobase per million) method using RSEM (Random-Sampling Empirical Myerson) quantification tool [52]. NOISeq method was used to screen differentially expression genes (DEGs) between two groups (saline *vs*. LPS) at 12, 36 and 72 h by using absolute fold change ≥ 2 and divergent probability ≥ 0.8 [53].

For the purpose of functional classification of genes, Gene Ontology (GO) database that offers an up to date database for terminology and comprehensive illustration of the characteristics of genes and gene products in a given organism species was used [54]. DEGs were annotated on the basis of three ontologies, namely, biological process, molecular function and cellular component. Similarly, Kyoto Encyclopedia of Genes and Genomes (KEGG) database (http://www.genome.jp/kegg) was used to discover the link between differential genes and various KEGG pathways and to determine functional pathway analysis [55].

### Statistical analysis

GraphPad Prism version 5.0 was used for statistical analyses. The data is shown as mean ± standard deviation (SD). Independent-samples *t* test was applied to calculate significant differences between groups in the same tissue regions. Bonferroni’s multiple comparisons test after one-way ANOVA test was applied to calculate statistical significance among multiple sample sets *versus* control samples. Differences were considered significant if *P* < 0.05 i.e., *P < 0.05, **P < 0.01 and ***P < 0.001.

### Availability of supporting data

All the relevant datasets supporting the findings of the current study have been provided in the article and its supporting information. The raw sequence reads (RNA-seq data) associated with this study have been submitted to the National Center for Biotechnology Information (NCBI) Short Read Archive (SRA) under accession code SRP093225.

## SUPPLEMENTARY MATERIALS TABLES







## Abbreviations

LPS: lipopolysaccharide
BF: bursa of Fabricius
RNA-seq: RNA sequencing
STm: *Salmonella* enterica serovar Typhimurium
TLRs: toll like receptors
HRH: horseradish peroxidase
PCNA: proliferating cell nuclear antigen
ssDNA: single stranded DNA
IPP: Image-Pro-plus
h: hour/hours
PBS: phosphate buffered saline
H&E: Hematoxylin and Eosin
IHC: Immunohistochemistry
SD: standard deviation
SABC: strept avidin biotin complex
DAB: 3,3’-diaminobenzidine
BSA: bovine serum albumin
NF-κB: nuclear factor-κB
IL: interleukin
G1: growth 1 phase
S phase: DNA synthesis phase
RT-qPCR: quantitative real time polymerase chain reaction
IOD: integrated optical density
TUNEL: terminal-deoxynucleotidyl transferase mediated nick end labeling
POD: peroxide
JNK: c-Jun NH_2_-terminal kinase
MAPK: mitogen-activated protein kinase
BPI: Bactericidal/permeability-increasing protein
AVD: avidin
CNN1: calponin 1
SEM: scanning electron microscopy
HMDS: hexamethyldisilazane
TdT: terminal deoxynucleotidyl transferase
PVDF: polyvinylidene difluoride
SDS-PAGE: sodium dodecyl sulfate polyacrylamide gel electrophoresis
GO: Gene Ontology
KEGG: kyoto encyclopedia of genes and genomes
