# Supplementary material for: Lipopolysaccharide induces acute bursal atrophy in broiler chicks by activating TLR4-MAPK-NF-κB/AP-1 signaling
Source: Oncotarget. 2017 Aug 5;8(65):108375–91. doi: 10.18632/oncotarget.19964 (PMC5752450; doi:10.18632/oncotarget.19964)
Supplement: Supplementary file 1 [file oncotarget-08-108375-s001.pdf]

Lipopolysaccharide induces acute bursal atrophy in broiler chicks by activating TLR4-MAPK-NF-κB/AP-1 signaling

SUPPLEMENTARY MATERIALS

For Supplementary Tables see in Supplementary Files
